# Identification of genes involved in xylose metabolism of *Meyerozyma guilliermondii* and their genetic engineering for increased xylitol production

**DOI:** 10.1186/s13568-020-01012-8

**Published:** 2020-04-20

**Authors:** Denise Atzmüller, Nadine Ullmann, Alexander Zwirzitz

**Affiliations:** 1 University of Applied Sciences Upper Austria, Stelzhamerstraße 23, 4600 Wels, Austria; 2 Austrian Biotech University of Applied Sciences, Konrad-Lorenz-Straße 10, 3430 Tulln, Austria

**Keywords:** *Meyerozyma guilliermondii*, Xylose, Xylitol, Xylitol dehydrogenase, Xylose reductase

## Abstract

*Meyerozyma guilliermondii*, a non-conventional yeast that naturally assimilates xylose, is considered as a candidate for biotechnological production of the sugar alternative xylitol. Because the genes of the xylose metabolism were yet unknown, all efforts published so far to increase the xylitol yield of this yeast are limited to fermentation optimization. Hence, this study aimed to genetically engineer this organism for the first time with the objective to increase xylitol production. Therefore, the previously uncharacterized genes of *M. guilliermondii* ATCC 6260 encoding for xylose reductase (XR) and xylitol dehydrogenase (XDH) were identified by pathway investigations and sequence similarity analysis. Cloning and overexpression of the putative XR as well as knockout of the putative XDH genes generated strains with about threefold increased xylitol yield. Strains that combined both genetic modifications displayed fivefold increase in overall xylitol yield. Enzymatic activity assays with lysates of XR overexpressing and XDH knockout strains underlined the presumed functions of the respective genes. Furthermore, growth evaluation of the engineered strains on xylose as sole carbon source provides insights into xylose metabolism and its utilization for cell growth.
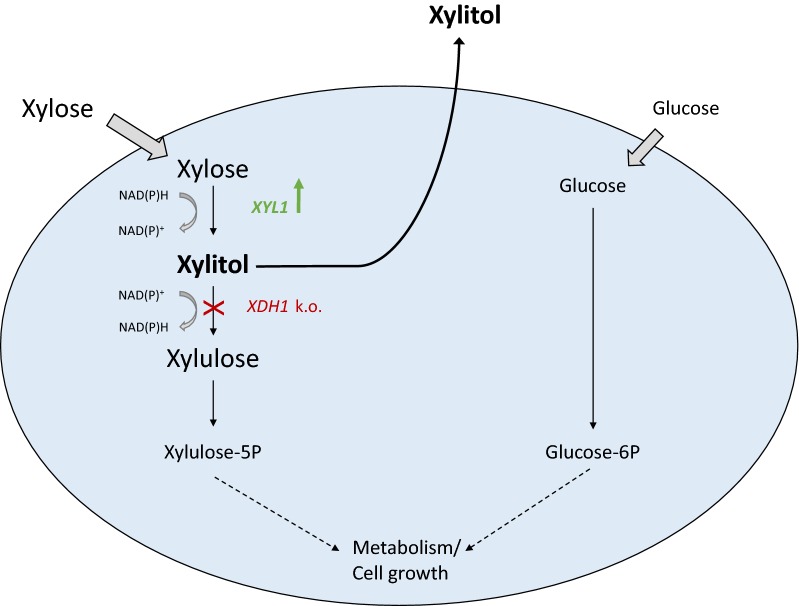

## Keypoints

- New xylose reductase (XYL1) and xylitol dehydrogenase (XDH1) genes were identified.
- *M. guilliermondii* was genetically modified to increase production of xylitol.
- Homologous overexpression of XYL1 and knockout of XDH1 promote xylitol production.

## Introduction

The interest on the five-carbon sugar alcohol xylitol has been constantly rising over the last years. This is not only because it is metabolized in an insulin independent manner and, therefore, is suitable for people suffering from diabetes, but also because of the rising interest in low-calorie diets (Ylikahri 1979). Xylitol tastes as sweet as sucrose whilst harboring about 40% less calories (Zacharis 2012). Furthermore, its anti-cariogenic properties make xylitol an interesting agent for the pharmaceutical industry (Mäkinen 1979).

Currently xylitol is produced chemically, which has some disadvantages in terms of energy demand, wastewater pollution and extensive purification requirements. The need of pure d-xylose as raw material for a nickel-catalyzed hydrogenation is one of the main cost rising factors, as this d-xylose has to be purified from mainly lignocellulosic material in a laborious and expensive way (Granström et al. 2007a; Rafiqul and Sakinah 2013).

In contrast, two biotechnological approaches are evolving over the last years: the enzymatic approach and the microbial process. For the enzymatic attempt, d-xylose is converted by purified xylose reductase (XR) from yeast. Herein the conversion is very efficient, but also has the drawback of using pure d-xylose as starting material (Kitpreechavanich et al. 1984; Neuhauser et al. 1998; Nidetzky et al. 2000). For the microbial xylitol production, the lignocellulosic starting material has to be pre-treated in certain ways, depending on the organism used, but the xylose does not have to be purified (Granström et al. 2007a; Rafiqul and Sakinah 2013; Albuquerque et al. 2014; Venkateswar Rao et al. 2015). This approach uses the fact that some microorganisms, bacteria as well as yeasts, are capable of producing xylitol in a fermentative way. Although some bacteria as *Corynebacterium* sp., *Enterobacter liquefaciens* or *Mycobacterium smegmatis* have been reported to produce xylitol (Winkelhausen and Kuzmanova 1998; Chen et al. 2010; Rafiqul and Sakinah 2013), yeasts are the more investigated candidates for microbial xylitol production (reviewed e.g. in (Granström et al. 2007b) and (Jeffries and Jin 2004)). Whilst there are several attempts of genetic modification of *Saccharomyces cerevisiae* to produce xylitol (Jeppsson et al. 2006; Dasgupta et al. 2017), other, non-conventional yeasts, like amongst others *Candida* sp., *Kluyveromyces* sp. or *Trichoderma reesei*, are natural producers of this sugar alcohol (Winkelhausen and Kuzmanova 1998; Chen et al. 2010; Venkateswar Rao et al. 2015), and are therefore probably more promising candidates for biotechnological production of xylitol.

**Fig. 1:**
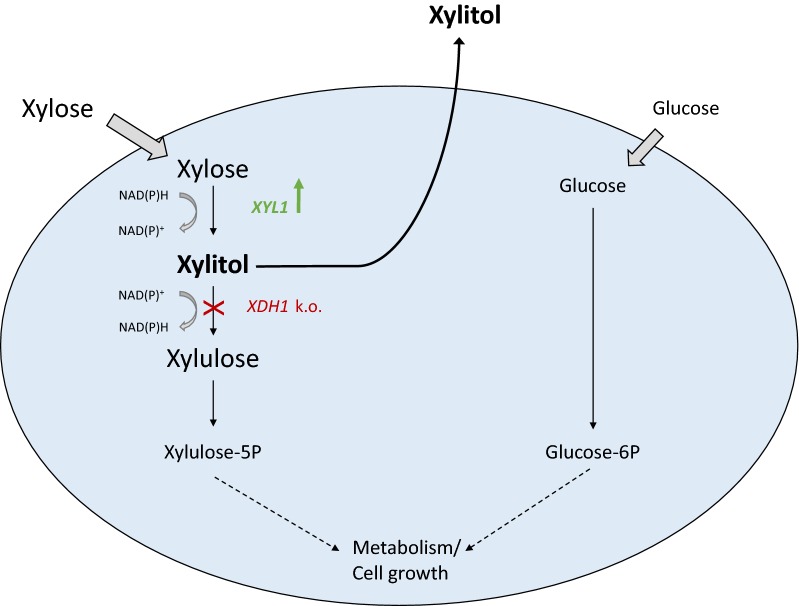
Illustration of xylose metabolism pathway. Xylose metabolism (part) of xylose-utilizing non-conventional yeasts (suggested in (da Silva et al. 2005)) Xylose metabolizing enzymes covered in this study are highlighted in green (*XYL1* = xylose reductase) and red (*XDH1* = xylitol dehydrogenase)

Generally, there are two ways microbes metabolize xylose. Bacteria use a one step process, catalysed by xylose isomerase (XI), for the direct conversion from xylose to xylulose (Karhumaa et al. 2007) whilst yeast and fungi use a two-step process (Fig. 1). The first step is the xylose reductase (XR; EC 1.1.1.307) or aldose reductase (EC 1.1.1.21) catalyzed conversion of xylose into xylitol, which then is further converted by xylitol dehydrogenase (XDH; EC 1.1.1.9) or l-iditol 2-dehydrogenase (EC 1.1.1.14) to form d-xylulose, which can be used in the pentose phosphate pathway (Granström et al. 2007b; Karhumaa et al. 2007; Rafiqul and Sakinah 2013), or by l-xylulose reductase (EC 1.1.1.10) or d-iditol 2-dehydrogenase (EC1.1.1.15) to form l-xylulose.

*Meyerozyma guilliermondii* (*anamorph: Candida guilliermondii*) is a non-conventional yeast, which produces xylitol naturally, and therefore has been under intense investigation for many years. Its relatively high tolerance to inhibitors which typically occur in hydrolysates of lignocellulosic material, which is a low-cost substrate of great interest, makes it an interesting organism for the use in biorefineries (Pereira et al. 2011). So far, most of the past work was focused on the optimization of the fermentation conditions or the substrate preparation (reviewed e.g. in (Mohamad et al. 2015)) but no genetic optimization regarding xylitol production in *M.* *guilliermondii* is available in the literature. One reason for the lack of research in this field may be, that the prerequisite for this intention, the availability of genetic tools for the organism, was just fulfilled by the work of different scientists in the last decade (Millerioux et al. 2011; Foureau et al. 2013; Defosse et al. 2014).

The encouraging potential of enhancing xylitol production genetically has been previously shown with other yeasts like *Candida* *tropicalis* or *Trichoderma* *reesei* (Ko et al. 2006b; Hong et al. 2014). Ko et al. (2006a, b) could reach a 97% xylose to xylitol conversion in their xylitol dehydrogenase gene disrupted *C.* *tropicalis* strain, compared to 67.2% of the wildtype (Ko et al. 2006a). Likewise, a knockout of XDH in *T.* *reesei* resulted in an increase from undetectable to 8.6 mM xylitol (Hong et al. 2014). In general, xylose metabolism in CTG clade family members, like *C.* *tropicalis*, *P.* *stipitis* or *M. caribbica* has been quite intensively studied (Amore et al. 1991; Byoung et al. 2006; Sukpipat et al. 2017). However, only limited data are available for *M. guilliermondii*: Handumrongkul et al. (1998) overexpressed a *XYL1* gene of *M. guilliermondii* ATCC 20118 in *P. pastoris* (Handumrongkul et al. 1998). Yet, genes responsible for xylose metabolism in *M. guilliermondii* have not been investigated within the organism itself. Consequently, the aim of this study was to identify and manipulate XR (*XYL1*) and XDH (*XDH1*) genes in *M.* *guilliermondii* and evaluate the impact on xylitol production.

## Materials and methods

### Strains and culture conditions

The *M. guilliermondii* strains used and generated in this study are listed in Table 1. KU141F1 was kindly provided by N. Papon (Foureau et al. 2013) and was used as parental strain for all genetic modifications in this study. KU141F1 and hence all strains derived thereof are originated from the reference strain *M. guilliermondii* ATCC 6260 (American Type Culture Collection, ATCC Manassas, USA). The strains were maintained on YNB(S)-agar (Yeast Nitrogen Base without amino acids 6.7 g l^−1^, Lactan; sucrose 20 g l^−1^; agar 20 g l^−1^) or YPS-agar (yeast extract 10 g l^−1^; peptone from casein 20 g l^−1^; sucrose 20 g l^−1^; agar 20 g l^−1^). Liquid cultures were prepared in YNB(S)- or YPS-media and incubated at 30 °C and 170 rpm in an orbital shaker. *Escherichia coli* XL1-blue was used as host for cloning. The wild-type and recombinant *E. coli* were cultured in Luria–Bertani (LB, Roth) liquid medium or on LB-agar plates, with or without ampicillin (100 mg l^−1^) as required, at 37 °C.

**Table 1:** *M. guilliermondii* strains generated and used in this study

Strain	Genotype	Plasmid	Refs.
ATCC 6260	wild type	–	–
KU141F1*	*ura5*, *ku70Δ*	–	(Foureau et al. 2013)
Ctrl (KU [EV])*	*ura5*, *ku70Δ*	[pG-URA5-P_ACT1_-T_TRP1_]	(Foureau et al. 2013)/This study
xdh∆::URA5*	*ura5*, *ku70Δ*, *xdh1∆::URA5*	–	This study
xdhΔ*	*ura5*, *ku70Δ*, *xdh1Δ (xdh1∆::REP)*	[pG-URA5-P_ACT1_-T_TRP1_]	This study
KU [XR]*	*ura5*, *ku70Δ*	[pU-XR]	This study
xdhΔ [XR]*	*ura5*, *ku70Δ*, *xdh1Δ (xdh1∆::REP)*	[pU-XR]	This study
xdh∆::XR*	*ura5*, *ku70Δ*, *xdh1∆::XYL1*	–	This study

* KU141F1 and hence all strains derived thereof are originated from the reference strain *M. guilliermondii* ATCC 6260

### Genetic modifications

The used primers are listed in Additional file 1: Table S1, plasmids in Table 2. For the knockout of the putative XDH gene, *XDH1* (PGUG_05726), 1000 bp upstream (primer P1 and P2) and 1000 bp downstream (primer P3 and P4) of *XDH1* were amplified by PCR and cloned up- and down-stream of the URA5-selection cassette of pGRU5R to generate pU-XDH-KO. The knockout cassette was amplified from the plasmid (primer P1 and P4) and transformed into *M.* *guilliermondii* KU141F1 to generate xdh∆::URA5. The successful homologous recombination was verified by PCR (primer S1, S2 and S3, shown in Additional file 1: Fig. S2). To eliminate the URA5-selection marker from the genome again, URA-blaster was performed as described by Foreau et al. (Foureau et al. 2012) by plating the mutants on media containing 1 mg ml^−1^ 5-FOA (5-Fluoroorotic acid) and 300 ng ml^−1^ Uridine, resulting in the xdhΔ strain.

**Table 2:** plasmids used and generated in this study

Plasmid	Purpose	Source/Refs.
pG-RU5R	Backbone for knockout plasmids, *URA5* marker	(Millerioux et al. 2011)
pG-URA5-P_ACT1_-T_TRP1_	Expression plasmid backbone for protein expression in *M.* *guilliermondii* (= empty vector control [pU-EV])	(Defosse et al. 2014)
pU-XDH-KO	Plasmid for genomic knockout of *XDH1* (PGUG_05726) with *URA5*	This study
pU-XR	Plasmid for the expression of *XYL1* (PGUG_00922) in *M.* *guilliermondii* (= [pU-XR])	This study
pU-XDH-XR-KO	Plasmid for genomic replacement of *XDH1* (PGUG_01218) by *XYL1* (PGUG_00922)	This study

In order to overexpress the putative XR-gene, *XYL1* (PGUG_00922), it was amplified by PCR from the genome of *M.* *guilliermondii* ATCC6260 (primer P5 and P6) and cloned under the control of P_Act1_, a constitutively active promoter into pG-URA5-P_ACT1_-T_TRP1_ to generate pU-XR. This was transformed into *M.* *guilliermondii* KU141F1 as described below and selected on YNB(S) agar plates to generate the strain KU [XR]. Further, pU-XR was transformed into the xdhΔ strain to generate the xdhΔ [XR] strain.

Moreover, the open reading frame of *XDH1* was replaced in the genome of *M.* *guilliermondii* KU141F1 by *XYL1*. Therefore, *XYL1* was amplified by PCR from the genome of *M.* *guilliermondii* KU141F1 (primer P9 and P10) and cloned into pU-XDH1-KO, generating pU-XDH1-XR-KO. The knockout cassette was amplified from the plasmid (primer P1 and P4) and transformed into *M.* *guilliermondii* KU141F1 to generate the strain xdh1∆::XR-URA5. The successful homologous recombination was verified by PCR (primer S1, S2 and S3). The URA5 selection marker was released by URA-blaster as described above, resulting in the xdh1∆::XR strain.

**Fig. 2:**
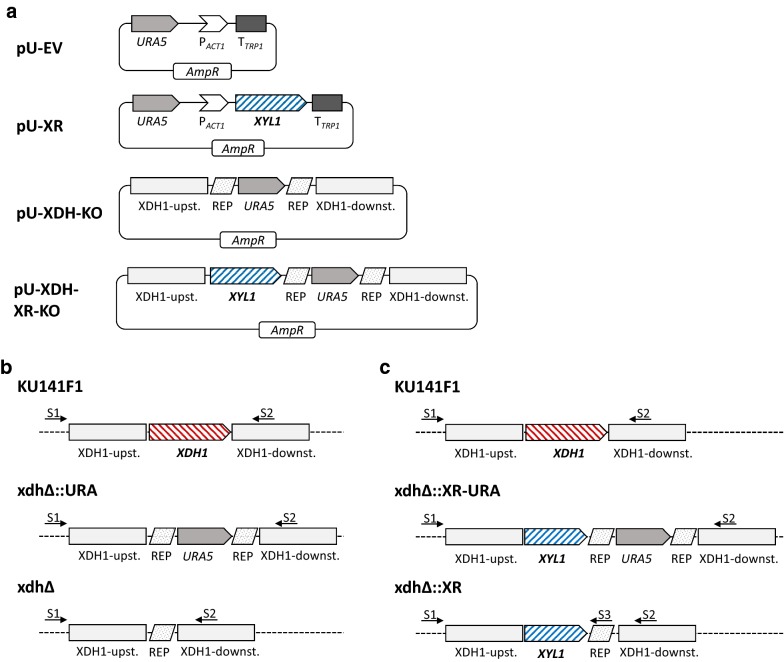
Schematic representation of the plasmids used and generated in this study, and the resulting genetic modifications. **a** shows the vectors used in this study: pU-EV is the empty vector used as control, pU-XR is used for the overexpression of the xylose reductase gene *XYL1*, pU-XDH-KO for the knockout of the xylitol dehydrogenase gene *XDH1*, and pU-XDH-XR-KO for genomic replacement of *XDH1* by *XYL1*. **b** Deletion of *XDH1* by homologous recombination of the selection cassette (xdh∆::URA) and after release of URA5 by Ura-Blaster (xdhΔ) in the parental strain *M. guilliermondii* KU141F1. **c** Replacement of *XDH1* by *XYL1* (xdh∆::XR-URA) and after release of URA5 by Ura-Blaster (xdh∆::XR) in the parental strain KU141F1; *AmpR:* Ampicillin resistance gene; *URA5*: selection marker for uridine-prototrophy, P_ACT1_: ACT1 promotor for constitutive expression of *XYL1*, T_TRP1_: Terminator of *XYL1* expression; REP: repetitive sequences for the use of the Ura-Blaster system to release URA5 selection marker; XDH1-upstr.: genomic sequence 1000 bp upstream of *XDH1*; XDH1-downst.: genomic sequence 1000 bp downstream of *XDH1;* S1, S2 and S3 indicate sequencing primer binding sites

Additionally, pG-URA5-P_ACT1_-T_TRP1_ without insert was transformed into KU141F1 (KU [EV]), xdhΔ (xdhΔ [EV]) and xdh∆::XR (xdh∆::XR [EV]) to serve as control strains. All the plasmids and resulting genetic modifications are illustrated in Fig. 2.

### Yeast transformation

The method for the transformation of *M.* *guilliermondii* was adapted from Boretsky et al. (2007).

Preparation of competent cells: The main culture was inoculated in 50 ml YPS + xylose (10 g l^−1^) and grown to an OD_600_ of approximately 0.5. The whole culture was harvested, washed and suspended in LiAc/TE-Buffer (lithium acetate 100 mM; TRIS–HCl 10 mM; EDTA 1 mM; pH 7.5). After incubation for 1 h at 30 °C and shaking, cells were harvested and re-suspended in LiAc/TE-Buffer to a concentration of approximately 5 × 10^9^ cfu ml^−1^ (OD 0.5 equals 2.4 × 10^7^ cfu ml^−1^). The suspension was spread into 50 µl aliquots for the transformation.

Transformation: 2–10 µg plasmid DNA or 2–2.5 µg linear DNA, 250 µl 50% PEG (polyethylene glycol) in LiAc/TE buffer and 5 µl herring sperm DNA (freshly denaturated at 95 °C for 10 min, chilled on ice) were added to the competent cells, mixed carefully and incubated at 30 °C for 30 min. After a heat shock (42 °C, 15 min) cells were centrifuged at 4 °C, re-suspended in 1 ml pre-warmed (30 °C) YPS and incubated for 3 to 5 h at 30 °C with shaking. Cells ware harvested, re-suspended in 100 µl 1 M sucrose, plated on selection plates and incubated at 30 °C for 2–3 days.

### Enzyme activity assay

The enzymatic activities of XDH and XR were analyzed after a modified protocol from Veras et al. (2017). Briefly, 10 OD units of an exponentially growing culture (cultivated in YNB(S) + 10 g l^−1^ xylose for 8 h) are harvested and washed with distilled water. Cells were lysed using Y-PER^®^ (Thermo Scientific, Vienna, Austria) according to the manufacturers protocol. The total protein in the cell free lysate was quantified by Roti^®^-Quant (Lactan, Graz, Austria) as described by the manufacturer.

The XR-reaction mix consisted of triethanolamine buffer (100 mM, pH 7.0), NADPH (0.2 mM) and xylose (350 mM). The XDH-reaction mix consisted of triethanolamine buffer (100 mM, pH 9.0), NAD^+^ (0.3 mM), xylitol (300 mM). The reactions were started by adding 1:10 crude cell lysate to the respective reaction mix and incubated at room temperature. Change of the absorbance at 340 nm, corresponding to co-factor conversion, was followed over 10 min. Enzyme activities (U mg_protein_^−1^) were calculated by using 6.22 ml (µmol cm)^−1^ as the molar absorption coefficient and 1 enzyme unit is defined as 1 μmol of cofactor reduction or oxidation per minute.

### Sugar and xylitol quantification

To analyze saccharides and xylitol, the culture samples were centrifuged at the indicated time points, and the supernatant was used for the quantification by HPLC, using a Jasco HPLC 2000 plus series (Biolab, Vienna, Austria) with an Aminex hpx 87 h column at 65 °C. H_2_SO_4_ (c = 5 mmol l^−1^) was used as eluent at an isocratic flow rate of 0.8 ml min^−1^. Data acquisition was performed with a refractive index detection. Data were analyzed with ChromPass (Version 1.8.6.1, Jasco Europe, Italy).

### Statistical analysis

All experiments were performed at least three times, each time in triplicates. Statistical analysis was performed with Prism v8.0.2—GraphPad Software, San Diego California, USA. Outliers were detected by a Grubbs outlier test and statistical significance was calculated via one-way ANOVA followed by a Tukey HSD test (xylitol yield) or students *t* test (enzymatic activity assays). Values are displayed as means with standard deviation of the mean. *p* < 0.05 = *, *p* < 0.005 = ** and *p* < 0.005 = ***.

## Results

To find the putative genes for XR and XDH in *M. guilliermondii*, investigations of the pentose and glucuronate interconversions pathway of *M.* *guilliermondii* 6260 (NCBI ref. gen. seq. AAFM00000000.1, assembly: GCF_000149425.1 from whole genome shotgun sequencing (Butler et al. 2009), were performed on the KEGG website (https://www.genome.jp/kegg-bin/show_pathway?pgu00040). This analysis revealed the enzyme activity EC 1.1.1.307 (d-xylose reductase) to PGUG_00922 of the *M.* *guilliermondii* ATCC6260 genomic scaffold 1 (NCBI ref. seq. NW_001809800.1). BLAST analysis revealed 94.32% sequence identity to the previously described XR of *M.* *guilliermondii* ATCC 20118 (alignment in Additional file 1: Fig. S1), which was 70.6% identical to XR of *S. stipitis* (Handumrongkul et al. 1998). Furthermore, activity EC 1.1.1.9 (d-xylulose reductase or NAD + -dependent xylitol dehydrogenase), which catalyzes the reaction from d-xylulose to xylitol and vice versa, was linked to PGUG_05726 of the *M.* *guilliermondii* ATCC6260 genomic scaffold 8 (NCBI ref. seq. NW_001809793.1). BLAST analysis revealed a 97.24% sequence identity to a previously described XDH of *M.* *caribbica* (Sukpipat et al. 2017).

### Cloning of *XYL1* and knockout of *XDH1*

PCR-amplification of the *XYL1* gene from *M.* *guilliermondii* ATCC 6260 genomic DNA with primers P5 and P6 yielded a 955 bp fragment, which was subsequently cloned into the expression vector pG-URA5-P_ACT1_-T_TRP1_ (Defosse et al. 2014). The successful cloning of the resulting expression plasmid (pU-XR) was confirmed by sequencing, then the plasmid was transformed into *M.* *guilliermondii* strains KU141F1 and xdh∆ (see Fig. 2 for a schematic representation of the plasmids and resulting genetic modifications). Transformants grown on selection medium without uridine allowed for selection of pU-XR carrying transformants.

To disrupt the putative *XDH1* gene, two approaches were applied: in a first attempt, the genomic site of PGUG_5726 (*XDH1*) was disrupted by the URA5 selection cassette (pU-XDH-KO). In the second approach, the *XDH1* gene was disrupted by the *XYL1* genomic sequence (pU-XDH-XR-KO). Thereby, a stable integration of an additional *XYL1* gene copy into the genomic DNA with a simultaneous disruption of *XDH1* was generated. The knockout of *XDH1* by *URA5* and *XYL1*, respectively, yielding the strains xdh∆ and xdh∆::XR, was screened by PCR (Fig. S2) and confirmed by sequencing.

### Enzymatic activity assessment of *XYL1* overexpressing and *XDH1* knockout strains

**Fig. 3:**
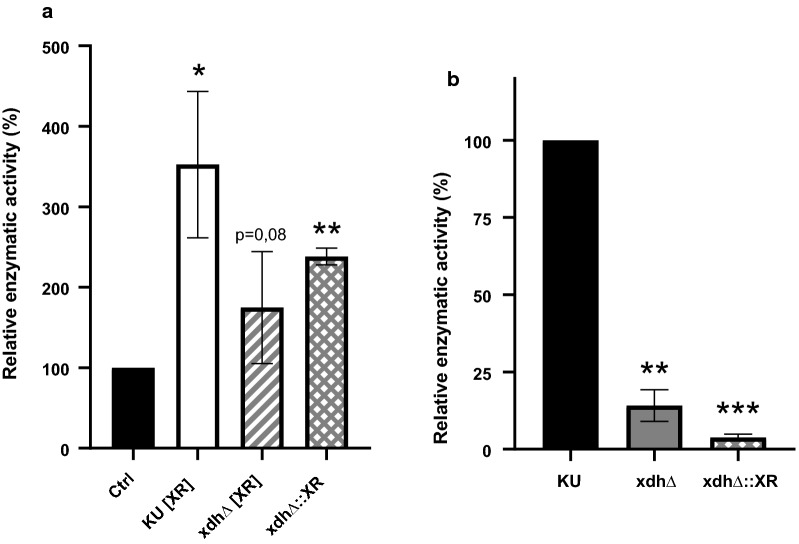
Enzymatic activity analysis of *M. guilliermondii* cell lysates. Measurement of co-factor conversion rates is displayed as **a** relative enzymatic XR-activity of lysates of KU [EV] (Ctrl), KU [XR], xdhΔ [XR] and xdh∆::XR; **b** relative enzymatic XDH-activity of lysates of KU, xdhΔ and xdh∆::XR; Displayed are mean values ± SD of at least 3 replicate experiments, each performed in triplicates. The control and parental strains were set to 100%. Asterisks indicate p-values (* = *p *< 0.05; ** = *p *< 0.005 and *** = *p*<0.001)

In order to characterize the putative *XYL1* and *XDH1* genes, enzymatic activity assays with crude cell lysates of strains overexpressing *XYL1* and strains with disrupted *XDH1* genes were performed. When xylose and NADPH were provided as substrates, lysates of KU [XR] indeed displayed 350% increased NADPH to NADP + conversion, see Fig. 3a. Lysates of xdh∆ [XR] displayed 175% and the xdh∆::XR strain 240% increased XR activity. Vice versa, lysates of xdh∆ and xdh∆::XR exhibited strongly decreased NAD + to NADH conversion when xylitol and NAD + were provided, see Fig. 3b. In ∆xdh only 14% and in xdh∆::XR only 4% of the parental strain’s XDH activity were remaining. This provides clear evidence that both genes actually encoded for the predicted enzymes, which converted the expected substrates and co-factors (xylose and NADPH for XR, and xylitol and NAD + for XDH).

### Growth evaluation of mutant strains

**Fig. 4:**
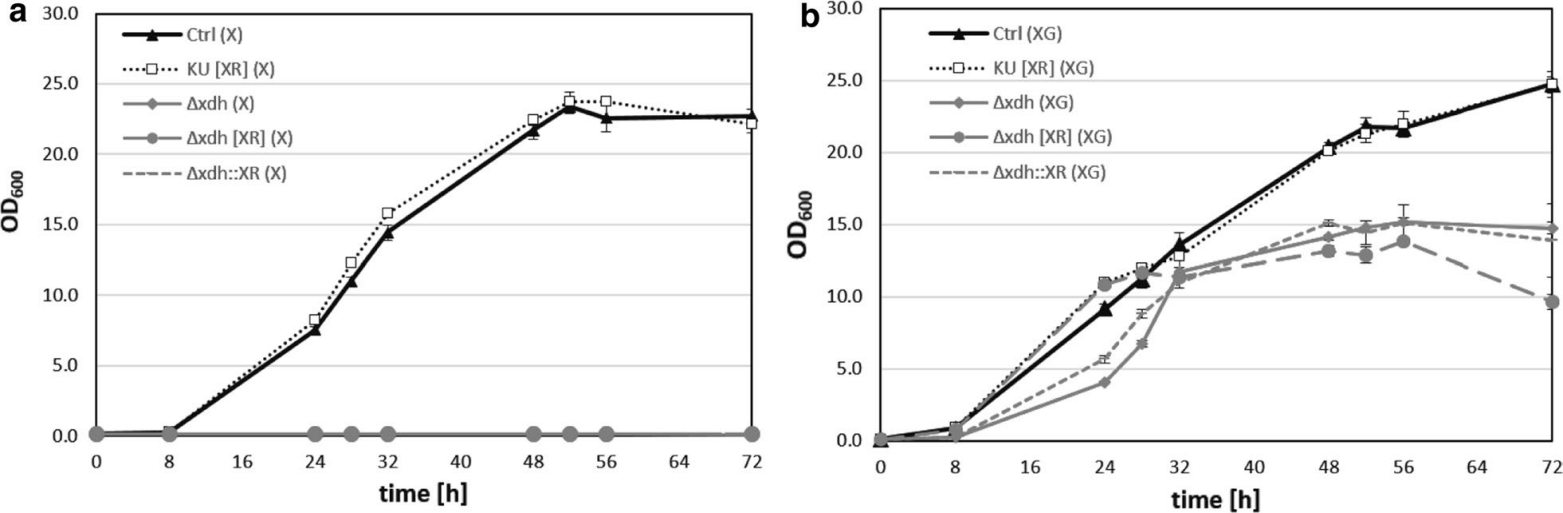
Growth evaluation of *M. guilliermondii* strains with xylose only or xylose + glucose as carbon source. Growth comparison of parental strain Ctrl (KU [EV]) (filled triangles) with mutant strains KU [XR] (open square), xdhΔ (filled diamond), xdhΔ [XR] (filled circle) and xdh∆::XR (dashed lines) in YNB media with **a** 20 g l^−1^ xylose (X) or **b** 20 g l^−1^ xylose and 20 g l^−1^ glucose (XG); Displayed are mean values ± SD of triplicates

To get an insight in growth behavior and xylose metabolism of the genetically engineered strains, growth in xylose containing media was assessed. As can be seen in Fig. 4, overexpression of *XYL1* did not markedly affect growth characteristics of *M. guilliermondii*. However, mutants with disrupted *XDH1* genes were not able to grow at all on media with xylose as the sole carbon source, see Fig. 4a. Supplementation with glucose as an additional carbon sources allowed growth of the various *XDH1* knockout strains (Fig. 4b). However, they grew notably slower and to considerably lower final densities compared to the control strain.

### Xylitol production of genetically engineered strains

**Fig. 5:**
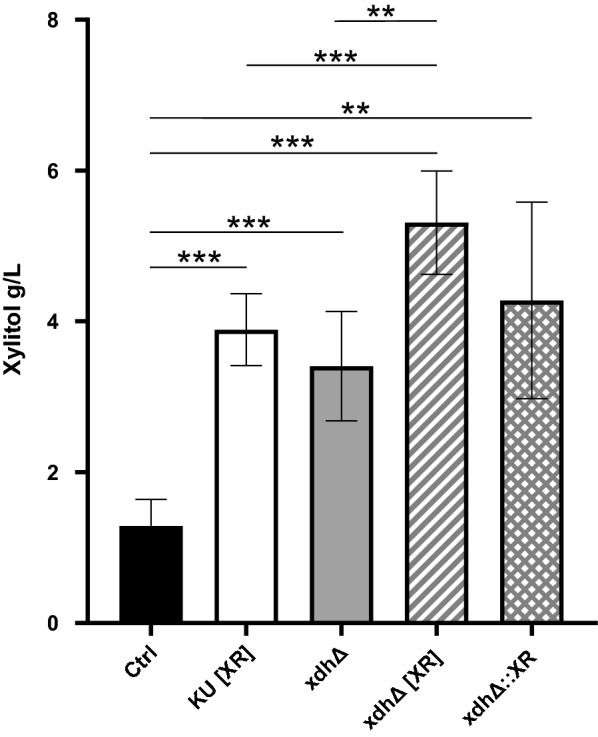
Xylitol production of *M. guilliermondii* mutant strains. Xylitol yield (in g l^−1^ as determined by HPLC) of KU [EV] (Ctrl); KU [XR]; xdhΔ, xdhΔ [XR] and xdh∆::XR after 72 h cultivation in YNB with 20 g l^−1^ xylose and 20 g l^−1^ glucose; Displayed are mean values ± SD of at least 3 replicate experiments, each performed in triplicates. Asterisks indicate p-values (** =* p *< 0,005 and *** = *p*<0,001)

Finally, the xylitol production of all strains generated during this project was analyzed in shake flask fermentations (shown in Fig. 5). Overexpression of *XYL1* resulted in a threefold increase in xylitol accumulation after 72 h, yielding 3.89 ± 0.47 g l^−1^ for KU [XR], compared to 1.29 ± 0.35 g l^−1^ for the control strain KU [EV]. Similarly, xdhΔ showed increased xylitol accumulation to comparable amounts, 3.41 ± 0.73 g l^−1^. Although overall growth of double mutant strains xdhΔ [XR] and xdh∆::XR was substantially lower than that of KU [XR], xylitol yield after 72 h was the highest of all the strains investigated in this study. xdhΔ [XR] produced 5.31 ± 0.69 g l^−1^ after 72 h of fermentation, resulting in a volumetric productivity of 0.074 g l^−1^ h^−1^ and a specific productivity of 0.27 g xylitol per g xylose. The knockout of *XDH1* by *XYL1* (xdh∆::XR) yielded 4.28 ± 1.30 g l^−1^ after 72 h of fermentation, resulting in a volumetric productivity of 0.060 g l^−1^ h^−1^ and a specific productivity of 0.21 g xylitol per g xylose. The yields and productivities of the above mentioned strains are summarized in Table 3.

**Table 3:** Yields (after 72 h growth on 20 g l^−**1**^ glucose and 20 g l^−**1**^ xylose) and productivities of the strains used in this study

Strain name	Yield (g l^−1^)	Vol. productivity (g l^−1^ h^−1^)	Spec. productivity (g xylitol per g xylose)
Ctrl (KU [EV])	1.29 ± 0.35	0.018	0.06
xdhΔ	3.41 ± 0.73	0.047	0.17
KU [XR]	3.89 ± 0.47	0.054	0.19
xdhΔ [XR]	5.31 ± 0.69	0.074	0.27
xdh∆::XR	4.28 ± 1.30	0.060	0.21

## Discussion

In this study, we provide data that allow interpretation of the function of two previously undescribed *M.* *guilliermondii* genes involved in xylose metabolism. On the one hand, we cloned and homologously overexpressed *XYL1* (PGUG_0922), which encodes for a putative XR. Indeed, overexpression of *XYL1* (KU [XR], xdhΔ [XR], xdh∆::XR) enabled higher xylitol accumulation in the medium, see Fig. 5. Moreover, lysates of *XYL1* overexpressing strains KU [XR] and xdh∆::XR showed significantly higher enzymatic activity on xylose with NAPDH provided as substrate (shown in Fig. 3a). Although the increase of co-factor conversion rates can also be observed in ∆xdh [XR] lysates, here the effect was not as outspoken as in the other XR overexpressing strains. It has been demonstrated that xylose reductases preferentially use NADPH as co-factor and that this specificity is determined by a conserved IPKS amino acid motif (Silva et al. 1996; Kostrzynska et al. 1998; Petschacher et al. 2005). PGUG_00922 of *M. guilliermondii* encodes for this sequence motif, highlighted in Additional file 1: Fig. S1, as well. Together, these data strongly support our hypothesis that *M.* *guilliermondii* PGUG_00922 is indeed a *XYL1* gene encoding for a XR that uses NADPH as a cofactor.

On the other hand, lysates of *XDH1* disrupted mutants (xdhΔ, xdh∆::XR) exhibited significantly lower enzymatic activity on xylitol with NAD + provided as a cofactor compared to lysates of the parental strain (KU), as shown in Fig. 3b, thereby providing evidence that the putative *XDH1* gene of *M.* *guilliermondii* PGUG_05726 is indeed a XDH that uses NAD + as a cofactor.

Mutants with disrupted *XDH1* genes (xdhΔ, xdh∆::XR) were not able to grow on xylose as sole carbon source (Fig. 4a). Conversely, the parental strain (Ctrl) grew equally well on xylose as a sole carbon source (Fig. 4a) compared to when glucose is present (Fig. 4b). These data strongly indicate that xylose metabolism is completely blocked in *XDH1* knockout strains. Obviously, its function (converting xylitol to d- or l-xylulose) could not be compensated by other enzymes, at least not in the experimental conditions applied. Hence, *XDH1* is probably the only enzyme in *M.* *guilliermondii* ATCC 6260 capable of processing xylitol and thus providing carbon for cell growth. Growth of *M.* *guilliermondii XDH1* knockout strains could be restored to a certain extent by supplementation with glucose (Fig. 4b). Correspondingly, the necessity of co-substrates for adequate growth of XDH-defective mutants has been demonstrated for xylose assimilating yeasts, like *C.* *tropicalis*, *P.* *stipitis, K. marxianus* and *D. hansenii* (Kim et al. 2001; Ko et al. 2006a; Pal et al. 2013; Zhang et al. 2014). Contrasting to our observations in *M. guilliermondii*, knockout of an XDH gene in *C. tropicalis* actually resulted in lower xylitol yield compared to the parental strain, due to growth impairment of the knockout strain (Ko et al. 2006a). Although, addition of glucose enabled growth of the *XDH1* knockout strains we generated, the parental strain still grew significantly better under these conditions. The major differences in the overall growth rate between wild type and *XDH1* defective strains can most probably be attributed to differences in the ability to utilize xylose. However, the parental strain also grew faster than xdhΔ and xdh∆::XR within the first 24–32 h. In this early growth phase only glucose is supposed to be used (shown by (Gurgel et al. 1998) and observed by us, data not shown). This might indicate that an intact xylose metabolic pathway seems to be required for realization of the full growth potential.

Overexpression of *XYL1* in addition to knockout of *XDH1* resulted in significantly increased xylitol levels (Fig. 5). Conversely, homologous overexpression of XR in an XDH-disrupted mutant of *T. reesei* only slightly increased total xylitol yield (Hong et al. 2014). However, in our hands xylitol production improves significantly only when overexpressed from a plasmid (xdhΔ [XR]). Simply supplying another genetic copy of *XYL1* at the *XDH1* locus (xdh∆::XR) only slightly increased the total xylitol yield. This might be due to different gene copy numbers or promotor activities of the expression vector and the *XDH1* locus.

The overall xylitol yields of the *M. guilliermondii* ATCC 6260 strain are intrinsically rather low compared to other xylitol producing yeasts or fungi (Pal et al. 2016; Dasgupta et al. 2017). Although total xylitol yield remains within the single-digit gram per liter range, it could be substantially enhanced by overexpression of XR and knockout of XDH in *M.* *guilliermondii* ATCC 6260. With the remarkable relative increase of about 500% of the double mutants in mind, it would be highly interesting to expand the strategy applied here, to other strains, like *M.* *guilliermondii* FTI 20037, that produce higher amounts of xylitol per se (Barbosa et al. 1988; Winkelhausen and Kuzmanova 1998; Das and De 2005). As shown for other *Candida* spp., optimization of media and fermentation conditions could certainly enhance this strains xylitol production rate (Horitsu et al. 1992). Further studies examining the underlying mechanisms of this relatively low xylitol productivity of *M.* *guilliermondii* ATCC 6260 and addressing the respective rate-limiting steps could provide further insights into xylitol production and xylitol metabolism in general. It would be interesting to see whether expression of heterologous XRs, improving xylose uptake or co-factor balancing could enhance this strains productivity.

*Meyerozyma guilliermondii* is a promising potential candidate that can grow on alternative substrates as carbon sources in biotechnological production processes (Carvalho et al. 2002; Mussatto et al. 2006; Pereira et al. 2011; Cassabarbosa et al. 2015; Hernández-Pérez et al. 2016; Martini et al. 2016; López-Linares et al. 2018). Insight into the genetic mechanisms underlying its xylose metabolism and its growth behavior will facilitate its future use as cell factory. Moreover, XRs of various species have been expressed in *S.* *cerevisiae* in order to enable fermentation of xylose and thereby improve biotechnological production of e.g. ethanol (Amore et al. 1991; Karhumaa et al. 2007; Watanabe et al. 2007; Petschacher and Nidetzky 2008; Bengtsson et al. 2009). The characterization of additional XRs will allow greater variability and fine-tuning of xylose conversion, also in heterologous expression systems.

## Supplementary information

**Additional file 1**. Additional figures and table.

## Data Availability

All relevant data are supplied within the manuscript and additional file.
