# Supplementary material for: Identification of genes involved in xylose metabolism of Meyerozyma guilliermondii and their genetic engineering for increased xylitol production
Source: AMB Express. 2020 Apr 20;10:78. doi: 10.1186/s13568-020-01012-8 (PMC7171046; doi:10.1186/s13568-020-01012-8)
Supplement: Supplementary file 1 — Additional file 1. Additional figures and table. [file 13568_2020_1012_MOESM1_ESM.docx]

Additional file to submission to:

**AMB Express**

Manuscript title:

**Identification of genes involved in xylose metabolism of *Meyerozyma guilliermondii* and their genetic engineering for increased xylitol production**

Author information:

Denise Atzmüller^1^, Nadine Ullmann^2^, Alexander Zwirzitz^1, *^

^1^ University of Applied Sciences Upper Austria, Stelzhamerstraße 23, 4600 Wels, Austria

^2^ Austrian Biotech University of Applied Sciences, Konrad-Lorenz-Straße 10, 3430 Tulln, Austria

* corresponding author: [alexander.zwirzitz@fh-wels.at](mailto:alexander.zwirzitz@fh-wels.at), phone: +43 50804 44479, fax: +43 5080 944463 ORCID: 0000-0002-0703-0005

Additional file 1: Figure S1


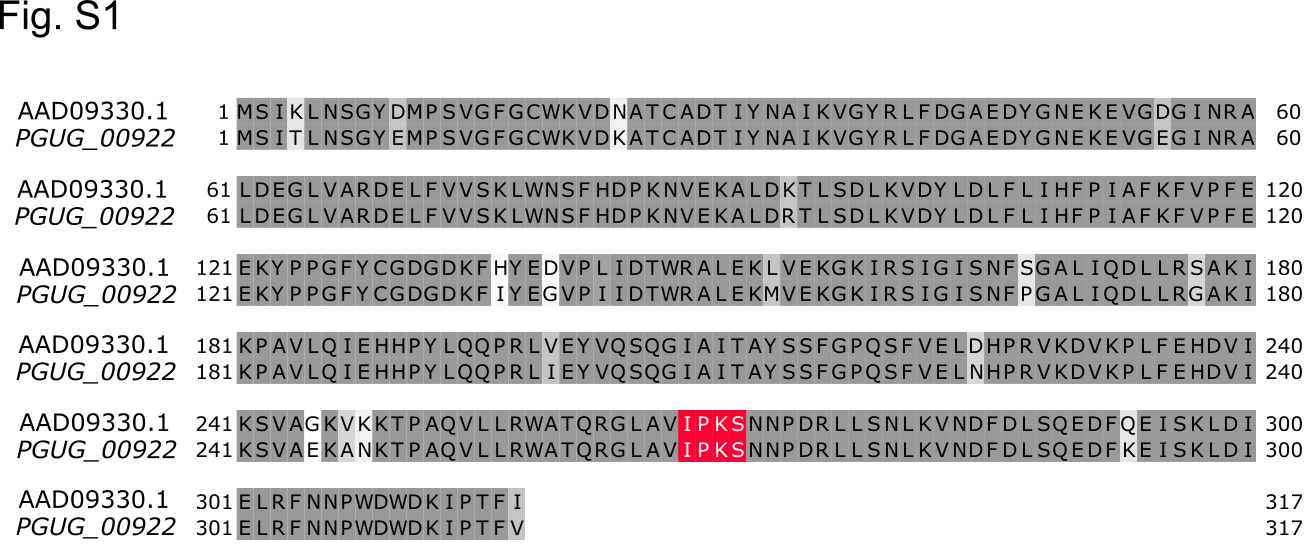


**Fig. S1 –** Amino acid sequence alignment with the previously identified *M. guilliermondii* *XYL1* gene

Amino acid alignment of *M. guilliermondii* *PGUG_00922* with the previously identified *XYL1* gene. Identical amino acids are marked in grey, similar amino acids in light grey, and different amino acids are unmarked (white). The conserved IPKS sequence motif that determines co-factor specificity is marked in red

Additional file 1: Figure S2


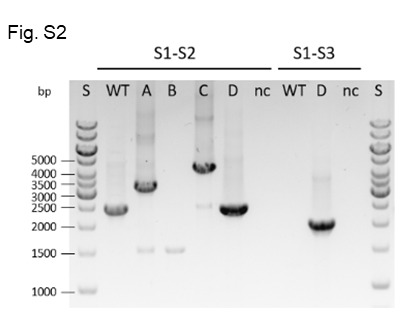


**Fig. S2 -** Confirmation of *XDH1* gene knockout

Verification of *xdh1*-knockout by PCR amplification with primer S1-S2 and S1-S3 from isolated genomic DNA; S1-S2 2.5 kb for WT (KU141F1), 3.3 kb for A (*xdh1∆*::URA5), 1.5 kb for B (*xdh1Δ*), 4.3 kb for C (*xdh1∆*::*XYL1*-URA5), 2.5 kb for D (*xdh1∆*::XR), no band for nc (negative control = without template); S1-S3 2.0 kb for D (*xdh1∆*::XR), no band for WT (KU141F1) and nc (negative control = without template) and; S: Size standard; The binding sites of primers S1, S2 and S3 are indicated in Fig. 2

Additional file 1: Table S1

Table S1 - primers used in this study

| **#** | **Name** | **Sequence (5’-3’)** | **Restriction sites** |
| --- | --- | --- | --- |
| P1 | *Apa*I-XDHup_F | AAT**GGGCCC**CCCGGGACTTGACCAGCCC | *Apa*I |
| P2 | XDHup-*Bam*HI_R | AAT**GGATCC**GAACAGAGTTCCTACAGCTATTACTACGGC | *Bam*HI |
| P3 | XDHdown-*Bgl*II_F | ATTA**AGATCT**TTCACTTCACCATCATGTCG | *Bgl*II |
| P4 | XDHdown-*Xho*I_R | AATA**CTCGAG**ATACGACAAATTATCTTGCCAGATGACTT | *Xho*I |
| P5 | *Pst*I-XR_F | AAT**CTGCAG**ATGTCTATTACTTTGAACTCAG | *Pst*I |
| P6 | XR-*Bam*HI_R | AAT**GGATCC**TTACACAAAAGTTGGAATCTTG | *Bam*HI |
| P7 | pU-KO_F | GTGAATTGTAATACGACTCAC | - |
| P8 | pU-KO_R | AGGAAACAGCTATGACCATG | - |
| P9 | *Bam*HI-XR_F | AAT**GGATCC**ATGTCTATTACTTTGAACTCAG | *Bam*HI |
| P10 | XR-*Nhe*I_R | ATT**GCTAGC**TTACACAAAAGTTGGAATCTTG | *Nhe*I |
| S1 | XDH1-KO-screen_F | CTACTTGGTGGCTTGAATATG | - |
| S2 | XDH1_seq_rev | CGACATGATGGTGAAGTGAA | - |
| S3 | REP_seq_R | GCCTCTCCACCCAAGCG | - |

**Table S1** **-** Primers used in this study

DNA sequence with highlighted restriction enzyme cleavage sites of the primers used in this study
